# Video Sensor-Based Complex Scene Analysis with Granger Causality

**DOI:** 10.3390/s131013685

**Published:** 2013-10-11

**Authors:** Yawen Fan, Hua Yang, Shibao Zheng, Hang Su, Shuang Wu

**Affiliations:** 1 Institution of Image Communication and Information Processing, Department of Electronic Engineering, Shanghai Jiaotong University, Shanghai 200240, China;E-Mails: hyang@sjtu.edu.cn (H.Y.); sbzh@sjtu.edu.cn (S.Z.); hmilyanjohn@sjtu.edu.cn (H.S.); wushuangseu@gmail.com (S.W.); 2 Shanghai Key Laboratory of Digital Media Processing and Transmission, Shanghai 200240, China

**Keywords:** video surveillance, scene analysis, topic model, point process, Granger causality

## Abstract

In this report, we propose a novel framework to explore the activity interactions and temporal dependencies between activities in complex video surveillance scenes. Under our framework, a low-level codebook is generated by an adaptive quantization with respect to the activeness criterion. The Hierarchical Dirichlet Processes (HDP) model is then applied to automatically cluster low-level features into atomic activities. Afterwards, the dynamic behaviors of the activities are represented as a multivariate point-process. The pair-wise relationships between activities are explicitly captured by the non-parametric Granger causality analysis, from which the activity interactions and temporal dependencies are discovered. Then, each video clip is labeled by one of the activity interactions. The results of the real-world traffic datasets show that the proposed method can achieve a high quality classification performance. Compared with traditional K-means clustering, a maximum improvement of 19.19% is achieved by using the proposed causal grouping method.

## 1. Introduction

Along with the developments of video surveillance systems, intelligent video analysis is a hot topic that has attracted significant attention, such as [1–4]. Scene analysis is one of the most important aspects of an intelligent video surveillance system. Automatically scene analysis, especially discovering activity interactions and temporal dependencies between them, is an active research area, but it remains a challenging problem due to complex video surveillance scenes with multiple activities occurring simultaneously. Currently, there are two main categories of approaches for discovering activity interactions and their temporal dependencies. The first category of approaches is based on probability inference. These methods typically use a Dynamic Bayesian Network (DBN), such as a Hidden Markov Model (HMM), or a probabilistic topic model (PTM) [5–8], such as Latent Dirichlet Allocation (LDA) [9], Hierarchical Dirichlet Processes (HDP) [10] and their extensions. The second category is data driven and based on the Granger causality [11] approach. Zhou *et al.* [12] perform the continuous-time Granger causality on pairs of trajectories to extract features for activity classification. Prabhakar *et al.* [13] perform the non-parameter Granger causality analysis on pairs of visual events, and demonstrate its effectiveness in exploring causal event sets without tracking. Based on the above-mentioned studies, Yi *et al.* [14] present a framework that models human actions using temporal causal relations of joint movements for human action classification. Nevertheless, both of their studies are applied to human action analysis.

Most of the aforementioned studies are based on tracking, which is limited to situations in which object tracking can be performed reliably. However, the successes of the topic model applied in complicated scenes analysis offers an advantage in that they can work directly on low-level motion features [5,6,8], e.g., optical flow, which avoids the unreliable detection and tracking. However, with respect to codebook generation, a fixed quantization is often used in these studies, which lose necessary resolution. Increasing the discretization resolution can reduce this loss but with a cost of increased training data and computation time.

**Figure 1.**
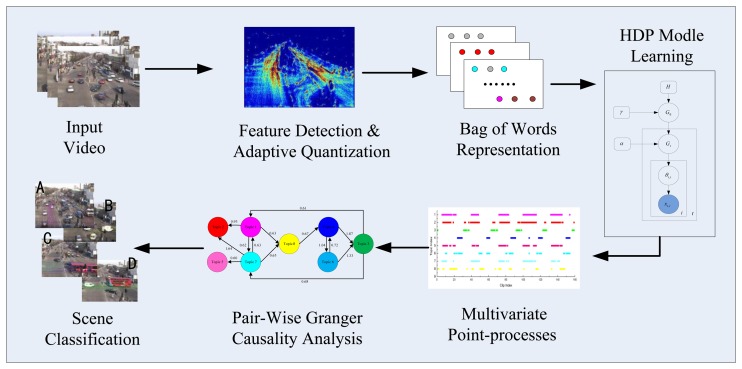
Flowchart of the proposed framework.

Our study builds on the success of the Granger causality applied to the analysis of visual events and takes advantage of the topic model. A flow chart of the proposed framework is shown in Figure 1. To create a temporal document from a video, low-level visual features are first detected and denoised. Then, a new method is proposed for codebook generation using adaptive quantization. Rather than using these words directly, low-level words are automatically clustered into topics (atomic activities) using the HDP model. Then, the dynamics of the visual activities are represented as a multivariate point-process [15]. Specifically, the temporal documents are created by counting the number of occurrences of the activities in each clip of the video. The pair-wise causal relationships between the atomic activities are then explicitly captured using the non-parametric Granger causality [16]. Based on the causal analysis results, the activity interactions and temporal dependencies are discovered, and a high quality classification performance is achieved.

The contributions of this study are two-fold: First, an activeness criterion-based method is proposed to determine the level of resolution of the optical flow quantization, and the low-level codebook is generated by using adaptive multi-scale quantization. Specifically, the window size of the location quantization is adaptive to the spatiotemporal characteristics. The second contribution is that the causality analysis is incorporated with the topic model to automatically explore the activity interactions and temporal links between them. Causal relationships between any pair of agents are established via the Granger causality statistic, from which we can obtain an improved classification performance.

The remainder of this paper is organized as follows. In Section 2, a statistical denoise method is proposed to obtain informative features. Then, a codebook with minimal size and adequate discriminative power is constructed based on the adaptive quantization. In Section 3.1, the video is interpreted as a point process based on the HDP model. The approach for the non-parametric Granger causality is explained in Section 3.2. In Section 4, we present the experimental results along with the analysis of the proposed approach for two real-world traffic sequences. Section 5 presents the conclusions and discusses future studies.

## 2. Feature Extraction and Adaptive Quantization

### 2.1. Feature Extraction and Denoising

In this section, optical flow is detected for video representation because it is typically more reliable in crowded scenes compared to the long-term trajectory in estimating various current activities. When the magnitude of the flow is smaller than a threshold, the flow is deemed unreliable and removed. Furthermore, the optical flow is denoised by determining whether it is informative for further analysis.

**Figure 2.**
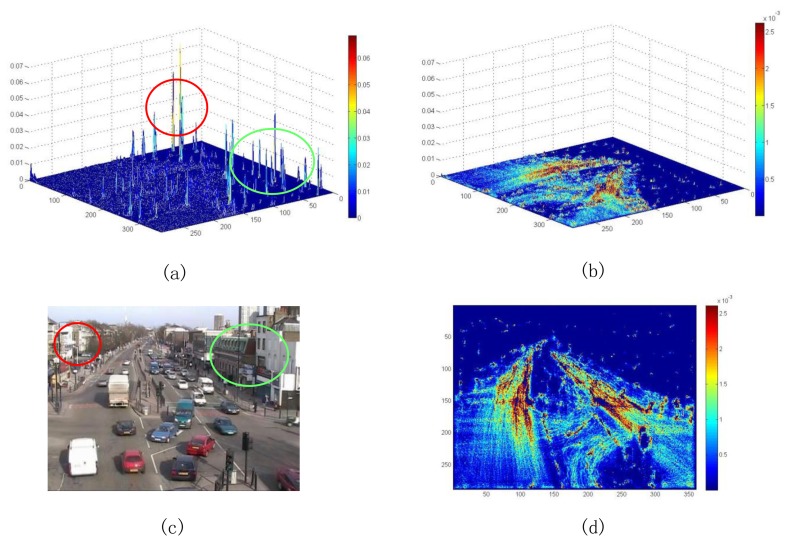
Optical flow spatial distribution. (**a**) 3D map without and (**b**) with statistical denoising; (**c**) scene image; (**d**) 2D map with statistical denoising.

For each pixel position, the total of optical flow vectors in the video sequence are counted. The result is a 2D matrix, and the matrix is divided by the total number of frames to obtain the probabilities. The probability for each position is a measure to determine whether this position is active (useful dynamic region). The pixel positions with extremely probabilities are deemed as a static region, while the pixel positions with very large probabilities are regarded as a noise dynamic region. Therefore, the optical flow vectors at these positions are discarded, which were not discarded by the magnitude threshold, because they are uninformative for further analysis. Figure 2 shows the comparison of the optical flow spatial distribution with and without statistical denoising for the intersection video sequence. It is evident that the statistical denoising allows the optical flow spatial distribution to be similar to the actual situation. For example, as shown in the Figure 2a, the probabilities become large in the areas labeled by red and green circles; in actuality, they are stationary regions corresponding to buildings (see Figure 2c).

### 2.2. Adaptive Quantization

In this section, a codebook is generated based on the denoised motion feature. To obtain a codebook, the previous methods often spatially divide the scene into non-overlapping grids (e.g., 5 × 5 and 10 × 10), and the direction is quantized into four or eight directions. This discretization necessarily causes spatial and directional resolution loss. The increasing discretization resolution can deduce this loss, but it also results in an increase in training data requirements and computation time. Furthermore, the fixed quantization does not consider the complexity of the motion in the actual scene.

To overcome the above limitations, we present a new method for codebook generation using adaptive quantization. The advantages of the proposed approach include maintaining a minimal size codebook with adequate discriminative power. Based on the observation, the optical flow spatial and directional distribution s may not be uniform, especially when observed over a significant length of time. Thus, a rough quantization will be used for the low activeness region. Furthermore, a fine quantization will be used for areas of the region in which motions are complex. First, an activeness criterion for a block is defined based on the flow density and diversity. The flow density of a block is defined as follows:

(1) $$F_{\text{density}}=\frac{N}{S}$$

where S is the area of the grid and N is the total number of optical flow vectors in this region. The density is then normalized by the maximum density value as follows: *D_max_*:

(2) $$S_{\text{density}}=\frac{F_{\text{density}}}{D_{\max}}$$

To compute the flow diversity for each grid, the optical flow directional histogram is first computed, and the histogram is normalized as a vector *H*(*d*_1_, *d*_2_, …, …*d_M_*). The number of bins in the histogram is *M*. The diversity of flows in the block is measured by the KL divergence (relative entropy) [17] between *H* and the union distribution as follows: $U=\overset{1}{\;}/\underset{M}{\;}$ (maximum entropy):

(3) $$D_{KL}(H\|U)=\sum_{i}^{M}d_{i}\log(\frac{d_{i}}{U_{i}})=\sum_{i}^{M}d_{i}\log(M\;\cdot \;d_{i})$$

Lastly, the diversity score of this block is calculated as follows:

(4) $$S_{\text{diversity}}=1-\frac{D_{KL}(H\|U)}{\log M}$$

The diversity score lies within [0,1], and a high score indicates that the direction distribution has more scatter. The activeness of the gird is measured by the combination of these two terms as follows:

(5) $$A=\mu S_{\text{density}}+(1-\mu )S_{\text{diversity}}$$

where parameter *μ* is the the prior mixture parameter for the two terms, and A ranges from 0 to 1. The first term on the right side in the above equation affects the flow density, and the second term affects the flow diversity In practical, the value of the parameter *μ* is determined by experience.

**Figure 3.**
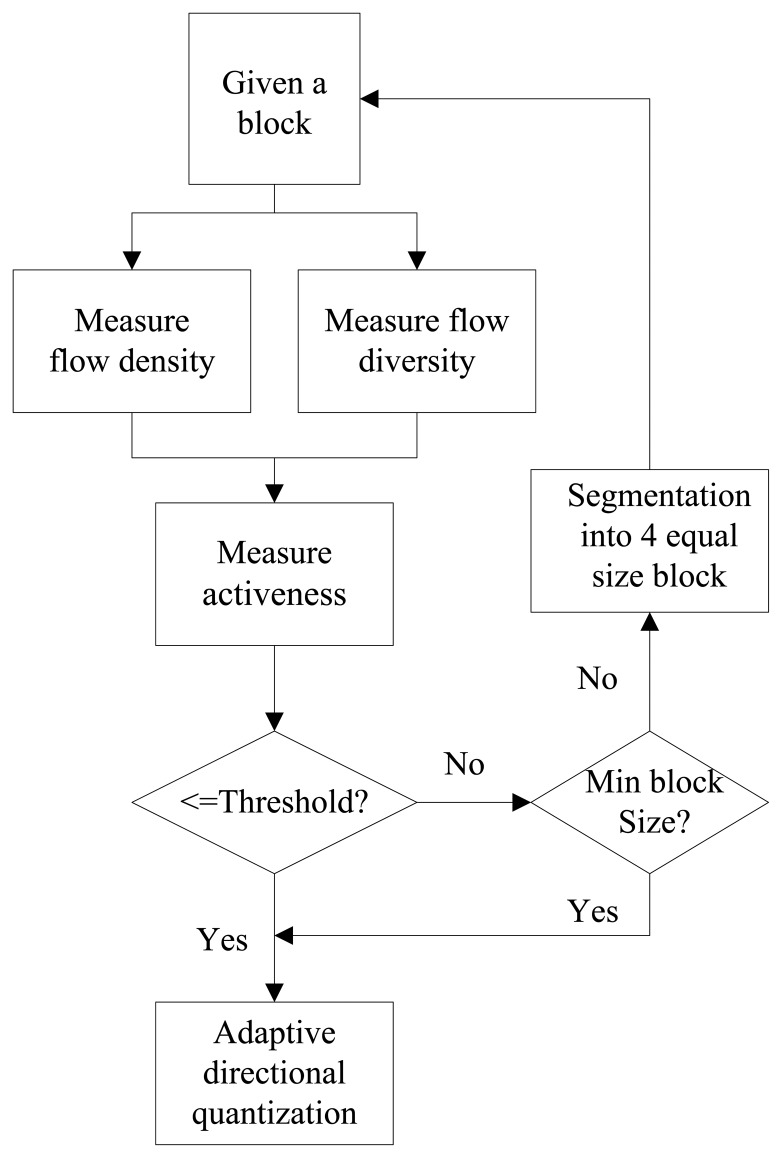
Algorithm of the adaptive multi-scale quantization.

**Figure 4.**
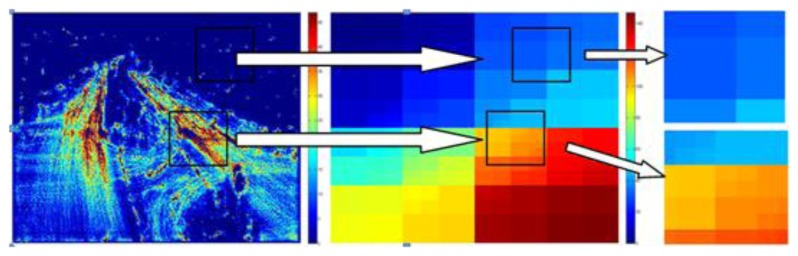
Illustration of the adaptive location quantization results for the intersection dataset.

Our approach of location quantization is similar to the quad-tree segmentation. A flow chart of the adaptive multi-scale location quantization is shown in Figure 3. The activeness of each initial block of size (*N* × *N*) is determined by comparing it to a threshold. If a block is sufficiently non-active, it is not divided further. However, if a block is active enough, the block is divided into four sub-blocks of identical size, the process is iterated on these four blocks individually. The process stops when each block is regarded as non-active or the minimum block size is reached. Figure 4 shows the illustration of the adaptive location quantization results for the intersection video sequence.

After location quantization, the direction is quantized based on the flow diversity. There are typically two degrees of directional quantization: four directions or eight directions. For one block, if its diversity score is larger than the predefined threshold, the direction in this block is clipped into eight. On the other side, the direction is clipped into four bins. After performing the spatial and directional quantization, a codebook is obtained with the size defined as follows,

(6) $$C_{\text{size}}=\sum_{i}^{L}d_{i}$$

where *L* is the total number of blocks, and *d_i_* is the size of the directional quantization in block i. The flow vectors from the sequence are mapped into one of the visual words. To establish a bag-of-words representation, a video is temporally clipped into non-overlapping clips, and its corresponding visual documents are composed with the words accumulated over its frames. The activities will be represented by co-occurring visual words.

## 3. Causal Analysis

### 3.1. From Video to Multivariate Point-Process

#### 3.1.1. Mid-Level Visual Words

**Figure 5.**
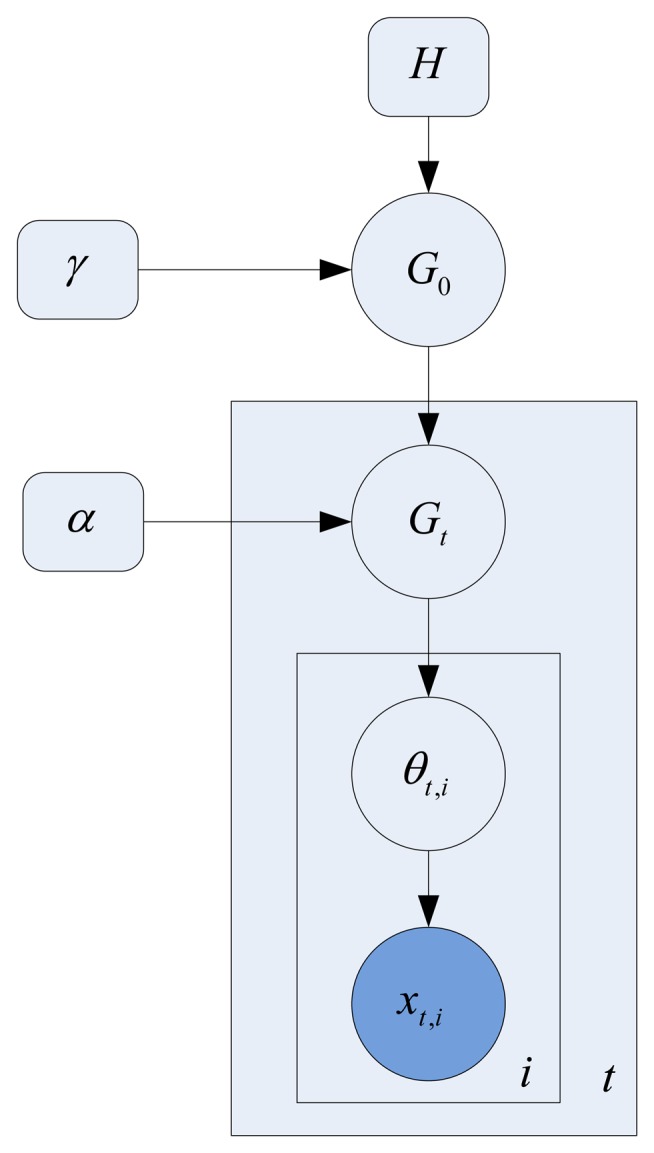
Hierarchical Dirichlet Process Model.

Although the size of the codebook is decreased by the adaptive quantization, its dimension is still high. In this section, the HDP [10] model shown in Figure 5 is used for clustering these low-level words into topics (atomic activities). The HDP is a nonparametric hierarchical Bayesian model. The advantage of using the HDP model is that it can automatically provide the number of discovered topics (atomic activities) that are deemed as mid-level visual words. Thus, the video can be represented in a more compact way.

There are two levels of the Dirichlet process (DP) in the HDP model, and the generative process is given as follows:

- In the first level, the DP generates a global random measure *G*_0_ with concentration parameter *γ* and base probability measure H as follows:
  (7) $$G_{0}|\gamma ,H∼DP\;(\gamma ,H)$$
  *G*_0_ can be formulated using the stick-breaking construction as follows:
  (8) $$\begin{array}{l} \pi_{k}^{'}|\gamma ,H∼\text{beta}\;(1,\gamma )\;\theta_{k}|\gamma ,H∼H\\ \pi_{k}=\pi_{k}^{'}\;\Pi_{l=1}^{k-1}\;1-\pi_{k}^{'}\;G_{0}=\sum_{k=1}^{\infty }\pi_{k}\delta_{\theta_{k}} \end{array}$$
- In the second level, the DP generates random measures *G_t_* for each clip *d_t_* with base probability measure *G*_0_ as follows:
  (9) $$G_{t}|\alpha ,G_{0}∼DP(\alpha ,G_{0})$$
  *G_t_* is a prior distribution of all the words in document *d_t_*, with only a subset of the topics in *G*_0_ active. It is formulated using the stick-breaking construction again as follows:
  (10) $$\begin{array}{l} {\hat{\pi }}_{k}^{'}|\alpha ,G_{0}∼\text{Beta}(1,\alpha )\\ \tau_{k}∼\text{Mult}(\pi_{1,}\pi_{2,}\ldots )\;{\hat{\theta }}_{k}|\alpha ,G_{0}=\theta_{T_{k}}\\ {\hat{\pi }}_{k}={\hat{\pi }}_{k}^{'}\;\Pi_{l=1}^{k-1}\;1-{\hat{\pi }}_{k}^{'}\;G_{t}=\sum_{k=1}^{\infty }{\hat{\pi }}_{k}\delta_{{\hat{\theta }}_{k}} \end{array}$$
- For the observed words *i* ∈ {1,…*N_j_*} in document *d_t_*:
  Draw a topic *θ_t,i_* ∼ *Multi*(*G_t_*);
  Sample a word *x_t,i_* ∼ *Multi*(*θ_t,i_*)

In the learning process, every low level word is assigned a topic identification. Furthermore a document (video clip) *d_t_* is represented by the mixture *G_t_* of topics. The discovered topics (atomic activities) will be directly used in creating a multivariate point-process in the following section.

#### 3.1.2. Multivariate Point-Process

For each video sequence, topics are detected and a multivariate point-process is generated by considering each topic *z_i_* as a point event. By applying the HDP inference on low-level word documents, the probability of topic *z_i_* in document *d_t_* can be estimated. The amount of occurrence of topic *z_i_* in the time interval (*t*, *t* + *dt*] is then defined as follows:

(11) $$dM_{i}(t)=M_{i}(t+dt)-M_{i}(t)=n_{t}\;\cdot \;p(z_{i}|d_{t})$$

where *dt* represents the time resolution; *M_i_*(*t*) denotes the number of topics in the interval (0, *t*]; *p*(*z_i_*∣*d_t_*) is the probability of topic *z_i_* in document *d_t_*; and *n_t_* represents the total number of visual words in the clip. The mean intensity of the process *M_i_*(*t*) is defined as *E*{*dM_i_*(*t*)} = λ*_i_dt*, and the zero-mean process *N_i_*(*t*) = *M_i_*(*t*) − λ*_i_dt* is considered as a point process. Therefore, these topics create a k-dimensional multivariate point-process *N*(*t*) = (*N*_1_(*t*), *N*_2_(*t*),…,*N_m_*(t))*^T^* for a video sequence.

### 3.2. Causal Analysis

#### 3.2.1. Nonparametric Granger Causality

In this section, a nonparametric estimation of Granger causality [16] for multivariate point processes is used. This method bypasses the autoregression (AR) model fitting. The calculation process is given as follows:

Given the multivariate point process, its spectral matrix is defined as follows:

(12) $$S(f)=\left(\begin{array}{ccc} S_{1,1}(f) & \cdots & S_{1,m}(f)\\ \cdots & \cdots & \cdots \\ S_{m,1}(f) & \cdots & S_{m,m}(f) \end{array}\right)$$

where off-diagonal elements represent the cross-spectrum, and diagonal elements represent the auto spectrum. The spectral matrix is estimated using the multitaper method [18], in which *K* Data tapers ${\left\{h_{k}\right\}}_{k=1}^{K}$ are applied successively to the *i*th topic, and the Fourier transform is taken as follows:

(13) $$\begin{array}{rrr} {\tilde{N}}_{i}(f,k) & = & \int_{0}^{T}h_{k}\exp(-2\pi \text{ift})dN_{i}(t)\\ & = & \underset{j}{\text{∑}}h_{k}(t_{j})\exp(-i2\pi ft_{j}) \end{array}$$

Lastly, the spectral matrix elements *S_ij_*(*f*) are estimated in the following function [19],

(14) $${\hat{S}}_{ij}=\frac{1}{2\pi KT}\sum_{k=1}^{K}{\tilde{N}}_{i}(f,k){\tilde{N}}_{j}{\left(f,k\right)}^{*}$$

The spectral matrix is factorized as follows: [20] :

(15) $$S(f)=T(f)\sum T{\left(f\right)}^{*}$$

where *T*(*f*) is the transfer function between processes and is the noise process covariance. After spectral factorizing, the Granger causality from *N_j_*(*t*) to *N_i_*(*t*) at frequency *f* is given by [21] as follows:

(16) $$G_{N_{j}\to N_{i}}(f)=\ln\left(\frac{S_{ii}(f)}{S_{ii}(f)-(\sum_{jj}-\sum_{ij}^{2}/\sum_{ii}){\left|T_{ij}(f)\right|}^{2}}\right)$$

Note that the measure is asymmetric, that is *G_Nj_*_→_*_Ni_* ≠ *G_Ni_*_→_*_Nj_*. A scalar measure of causality between processes N*_j_*(*t*) and N*_i_*(*t*) can be obtained by integrating Equation (16) with respect to the frequency, and the causal score can be obtained as follows:

(17) $$C(j,i)=\sum_{f}G_{N_{j}\to N_{i}}(f),\forall i\neq j$$

where *C*(*i*, *i*) = 0, ∀*i*.

#### 3.2.2. Causal Graph

To discover the interaction event sets, the pair-wise causal scores are first thresholded. The threshold is computed using an empirical null-hypothesis testing framework [22]. Finally, the Granger causality is represented by a directed graph, where nodes denote topics and edges denote the causal relations between them. Generally, the relations between two topics (e.g., topic 1 and topic 2) may have four cases:

- *Topic* 1 → *Topic* 2 : Topic 1 drives topic 2.
- *Topic* 1 ← *Topic* 2 : Topic 2 drives topic 1.
  In both of the above cases, we define these two topics as temporal causal topics.
- *Topic* 1 ⇆ *Topic* 2 : There is a bidirection causal relation between these two topics yielding the conclusion that the two topics are reciprocally coupled. We define them as temporal concurring topics.
- *Topic* 1 × *Topic* 2 : There is no direct causal relation between these two topics.

## 4. Experiments and Discussions

In this section, the proposed algorithm was tested on three public video sequences: a street intersection dataset (360 × 288, 25 fps, 1 h), a roundabout dataset (360 × 288, 25 fps, 1 h), and a subway platform dataset (360 × 288, 25 fps, 40 min). Both the street intersection dataset and the roundabout dataset are traffic videos governed by traffic lights in a certain temporal order selected from the QMUL dataset. Thus, the sequence of activities exhibits spatial-temporal periodicity. Typically, there are several flows at a time, and each flow may last for a period. The subway platform dataset is from the UK Home Office i-LIDS dataset and has been reedited. This dataset is significantly different from the other two datasets. It is captured indoors and mainly features humans and trains. The camera was mounted significantly lower and closer to the objects. The typical behaviors in this scene include people leaving or approaching the platform, and people getting on or off the train. We assess the proposed method with both qualitative and quantitative evaluations. First, we demonstrate that our analysis can explore the activity interactions and temporal causal relationships. Then, we quantitatively evaluate the results of casual analysis through the task of scene classification.

### 4.1. Activities Analysis

In this section, we apply our approach to extract the activities and then the activities are represented by multi-point processes. Each video was temporally segmented into 3-s long clips. The optical flows were computed and denoised. Furthermore, the optical flows were mapped into words based on the adaptive quantization. Finally, the HDP was applied to learn a generative model of video clips. During the learning process, this model effectively clusters concurring visual words into topics (activities). Twenty-one topics were automatically discovered by the HDP model for the intersection dataset, 26 topics were discovered for the roundabout dataset and 29 topics were discovered for the subway platform dataset. The topics are represented by different colors and will be further analyzed to search for temporal causality.

**Figure 6.**
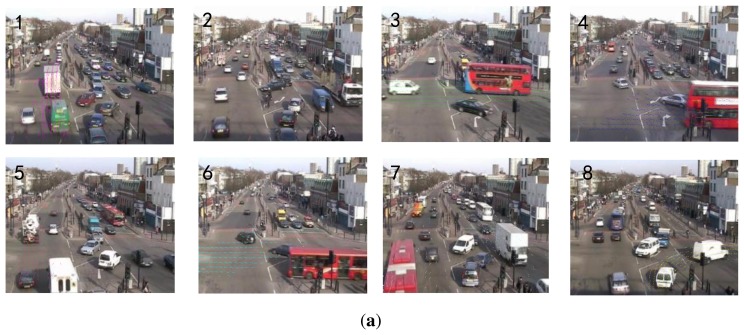

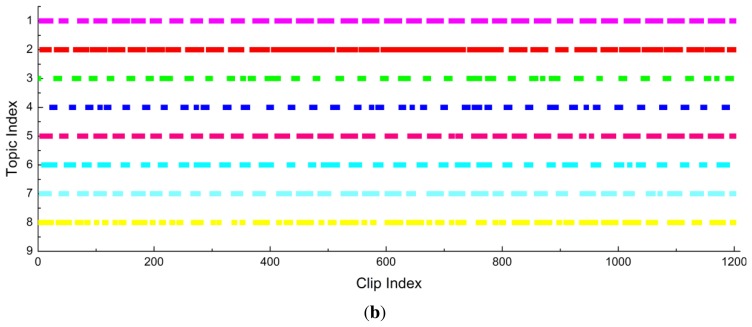
The experimental results on the intersection dataset. (**a**) Top: illustration of the top 8 topics; (**b**) Bottom: the timelines of the top 8 topics.

Figure 6a shows the motion distributions of the top eight topics (sorted by size) that explain at least 5% of all observations for the intersection dataset. Topics 1 and 5 describe vehicles moving upward along different lanes. Topic 8 describes vehicles turning right. Topics 2 and 7 describe vehicles moving downward but at different zones. Topic 3 describes the horizontal traffic flow from left to right. Topics 4 and 6 describe the horizontal traffic flow from right to left. Topic 6 may be shared by several activity interactions. Based on these topics, the eight point-processes are constructed and shown in Figure 6b.

**Figure 7.**
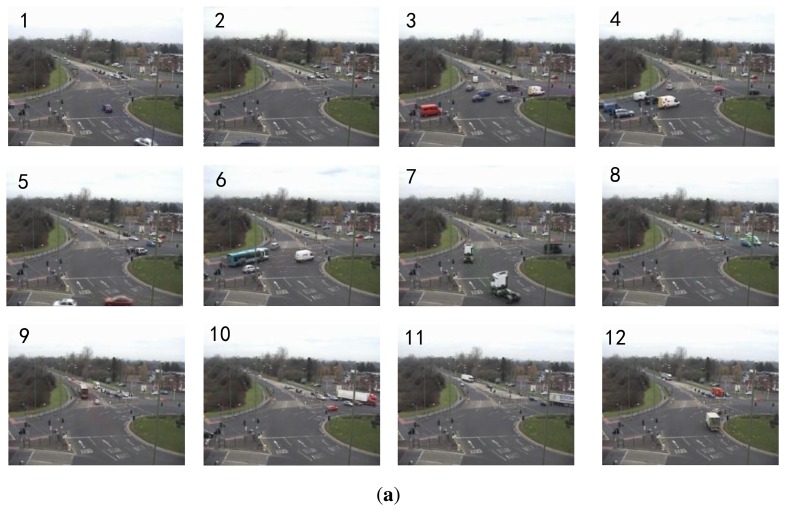

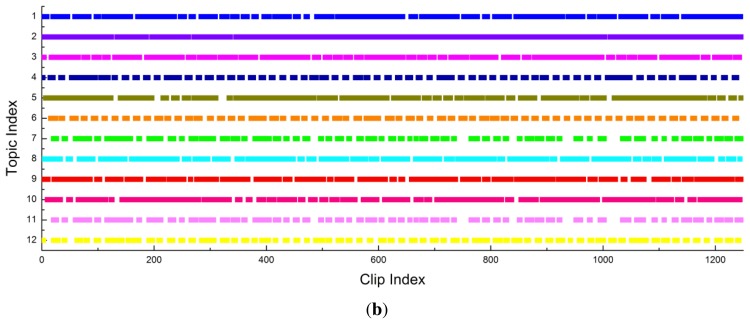
The experimental results on the roundabout dataset. (**a**) Top: illustration of the top 12 topics; (**b**) Bottom: the timelines of the 12 topics.

For the roundabout dataset, the motion distributions of the top 12 topics (sorted by size) that explain at least 3% of all observations are shown in Figure 7a. Topics 1,7 and 9 describe the upward traffic flows along different lanes. Topics 3, 4 and 6 describe the horizontal traffic flows from left to right at different zones. Topics 2 and 5 represent the horizontal traffic flows from right to left in near field. Topics 8, 10 and 11 represent the downward and leftward traffic flows in the far field. Topic 12 describes rightward turning. Figure 7b shows the corresponding twelve point-processes.

**Figure 8.**
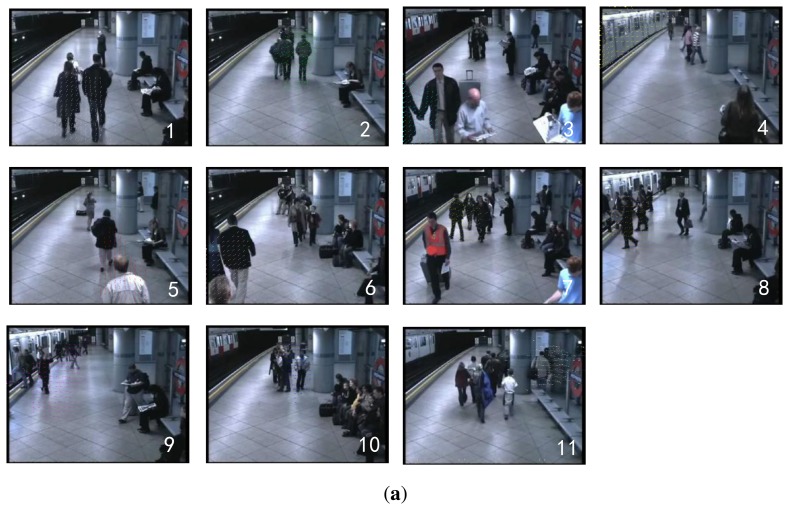

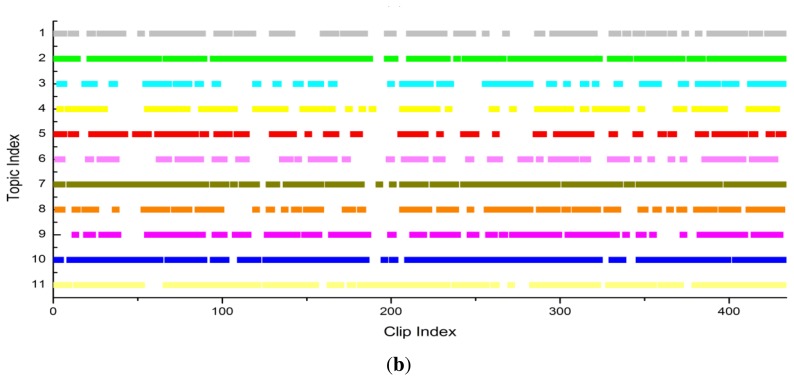
The experimental results on the subway dataset. (**a**) Top: illustration of the top 11 topics; (**b**) Bottom: the timelines of the 11 topics.

For the subway platform dataset, we select the top eleven topics (sorted by size) that explain at least 4% of all observations. As shown in Figure 8a, topics 1, 2, 5, 6 and 11 describe the people leaving the platform. Topic 4 describes the motion of the train. Topics 8 and 9 represent the people getting on and off the train respectively. Topics 3, 7 and 10 represent the people approaching the platform. Figure 8b shows the corresponding eleven point-processes.

### 4.2. Granger Causality Analysis

**Figure 9.**
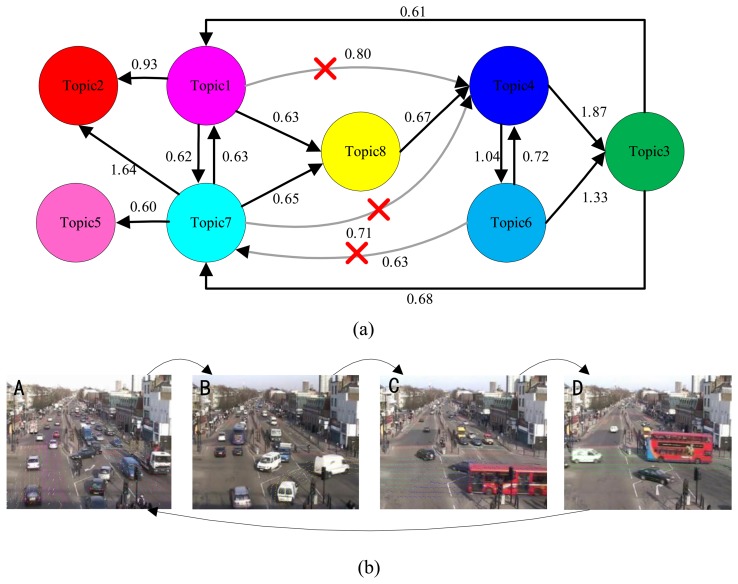
Granger causality approach applied to the intersection dataset. (**a**) Top: visualization of the temporal causal analysis. The connection between topic 6 and topic 7 is spurious because of the mediated influence from topic 3. Similarly, the connections between topic 1,7 and 4 are also removed; (**b**) Bottom: a scene with traffic lights. Four states are automatically found.

**Figure 10.**
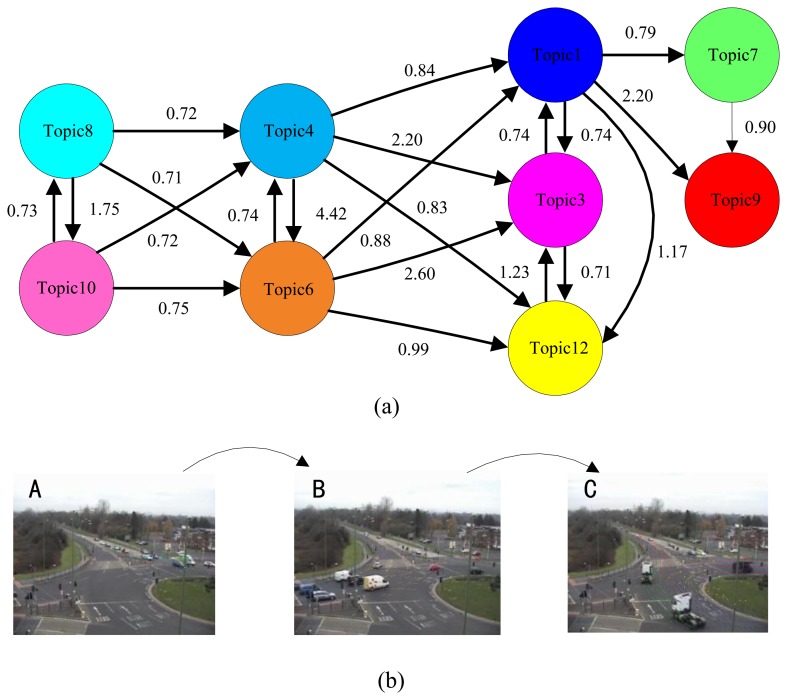
Granger causality approach applied to the roundabout dataset. (**a**) Top: visualization of the temporal causal analysis; (**b**) Bottom: a scene with traffic lights. Three states are automatically found.

**Figure 11.**
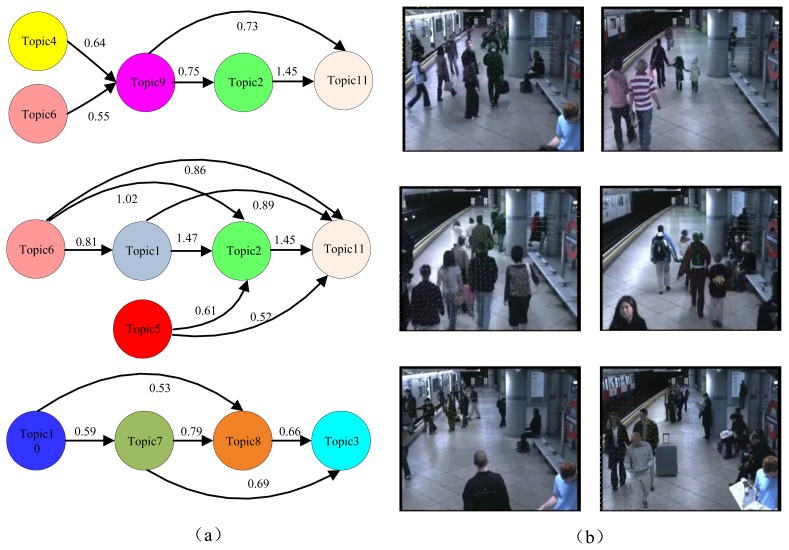
Granger causality approach applied to the subway dataset. (**a**) Left: visualization of the temporal causal analysis; (**b**) Right: six motion patterns are automatically found.

To automatically discover the activity interactions and temporal causal relationships between the topics, the analysis of Granger causality analysis was applied to the processes of Figures 6, 7 and 8. The results are shown in Tables 1, 2 and 3. Causal scores less than the threshold (0.6 for the intersection dataset, 0.7 for the roundabout dataset, and 0.5 for the subway platform dataset) are deemed as a no causal relationship. Then, the causal matrixes are then interpreted as directed causal graphs. As shown in Figure 9a, Figure 10a, and Figure 11a, nodes represent topics and edges denote detected pair-wise relationships. It can be observed in Table 2 that topics 2, 5 and 11 have no causal relationships with the other topics. This trend indicates that these three topics occur independently of the other topics.

In Figure 9a, a connection from topic 6 to topic 7 is observed, while another indirect connection from topic 6 to topic 7 through topic 3 is observed. To distinguish the direct causal influence from the indirect causal influence, the conditional Granger causality is computed. When topic 3 is in the on condition, the causal score from topic 6 to topic 7 (0.56) falls below the threshold value (0.6). Therefore the connection between topic 6 and topic 7 is spurious because of the mediated influence from topic 3. This connection is removed in the Figure 9a. Similarly, the connections between topic1, 7 and 4 are also removed.

Based on the definitions of the relations between two topics (see Section 3.2.2.), we initially find two temporal concurring topic sets: topics {1 7} and topics {4 6}. Then a main global temporal topic cycle is discovered, *i.e.*, topics {1 7} → {8} → {4} → {3} → {1 7}. Meanwhile, topics 1 and 7 co-cause topic 2, and topic 7 also causes topic 5. However, topics 2 and 5 have no causal influences on the other topics. In conclusion, four activity interactions (states) are found:

- State A: topics {1 7 2 5};
- State B: topics {8};
- State C: topics {4 6};
- State D: topics {3};

Furthermore, then the temporal causal relationship between them is explored, as follows:

{1 7 2 5} → {8} → {4 6} → {3} → {1 7 2 5}.

The visualizations of the temporal groupings are shown in Figure 9b. It is evident that the Granger causality results can identify the traffic light cycle governing the scene. Thus, four states are automatically founded.

Compared to the intersection dataset, the scene from the roundabout dataset is more complex and the video sensor is mounted significantly further from the objects. In Figure 10a, there are three sets of concurring topics: topics {8 10}, topics {4 6} and topics {1 3 12}. In conclusions, these topics are grouped into three activity interactions (states):

- State A: topics{8 10};
- State B: topics{4 6};
- State C: topics{1 3 7 9 12};

The temporal relationship between them is given as follows:

{8 10} → {4 6} → {1 3 7 9 12}.

We determined that the proposed approach failed to detect the relationship between the State C and State A because State A and State C would occasionally occur simultaneously. Therefore, the complete traffic light cycle is not discovered. The visualizations of the temporal groupings are shown in Figure 10b. Thus, three states are automatically founded.

Compared with the above two scenes regulated by traffic lights, the global temporal order in the subway platform scene is not obvious. However, the local temporal orders of the activities are well identified. To clarify we construct three causal graphs, as shown in Figure 11a. Furthermore, the corresponding six motion patterns are illustrated in Figure 11b.

- State A: topics {4 → 9 → 2 → 11};
- State B: topics {6 → 9 → 2 → 11};
- State C: topics {6 → 1 → 2 → 11};
- State D: topics {5 → 2 → 11};
- State E: topics {1 → 7 → 8};
- State F: topics {1 → 7 → 8 → 3};

State A represents the behavior where people get off the train and leave the platform after the train arrives. States B, C and D represent the motion of people leaving the platform along different lines. State E represents the behavior of people approaching the platform and getting on the train. State F represents the behavior of people traveling through the platform.

### 4.3. Scene Classification

To objectively measure the performance of the proposed approach, we use a scene classification task. We select the intersection and roundabout datasets for this experiment. Based on the causal analysis results, a causal grouping method is proposed for the scene classification. After the use of the HDP model as a feature dimension reduction step, a distribution over topics is associated with each clip. For causal grouping, we choose the same dominating topics (see Section 4.1) distribution to represent the clips. Then, the largest topic is selected for each clip, and we automatically predict the state in which it belongs. Finally, the short video clips are grouped into different classes. The classification performance of the proposed casual grouping method is compared with the K-means clustering method. The K-means method (Bhattacharyya distance is adopted) directly clusters video clips based on the topic distributions as feature vectors. The number of cluster centers of K-means is set to the same as the causal grouping. After clustering, each cluster is manually identified to determine in which class it belongs. In each case, the results are quantified in terms of the overall classification accuracy. To evaluate the classification performance, a ground truth is created by manually labeling the whole video clips into different typical interactions.

Table 4 shows the comparisons of the classification accuracy for the intersection dataset with different quantization resolutions. For constant quantization, the optical flow directions are quantized into four bins. For location quantization, there are five different resolutions(4 × 4, 8 × 8, 16 × 16, 32 × 32 and 64 × 64). In this experiment, the K-means technique is used as the classification method. It is evident that with the adaptive quantization, the size of the codebook decreases while the classification accuracy improves.

Tables 5 and 6 show the comparisons of the classification accuracy between the k-means clustering method and our causal grouping method for the intersection and roundabout datasets respectively. For the intersection dataset, the performance of the proposed approach is superior to the K-means clustering in the case of classes A, C and D. However, in the case of class B, the causal grouping is inferior to the K-means clustering. Similarly, for the roundabout dataset, the causal grouping method shows a significantly better performance than the K-means clustering method, except for class A. However, for the overall classification accuracy, the proposed approach always produces superior performance compared to the k-means clustering. Particularly, as seen from Table 6, the K-means clustering only obtains a 70.20% overall classification accuracy, while the causal grouping method is 89.39%.

**Figure 12.**
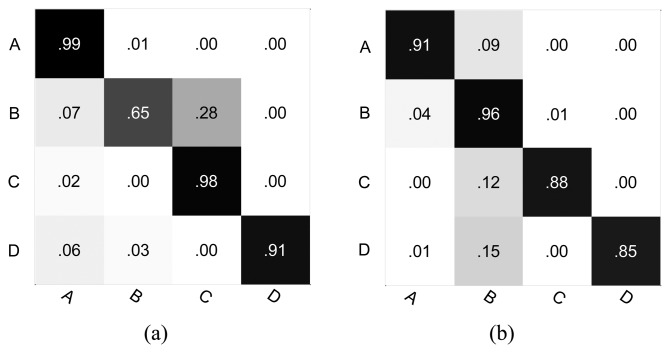
The confusion matrix for the intersection dataset (**a**) Causal grouping; (**b**) K-means clustering.

**Figure 13.**
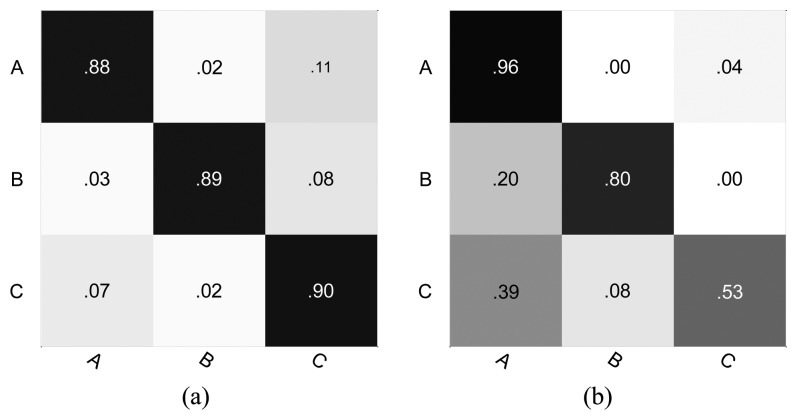
The confusion matrix for the roundabout dataset. (**a**) Causal grouping; (**b**) K-means clustering.

As shown in Figures 12 and 13, the full classification performance is also evaluated using a normalized confusion matrix. Our method results in a high true positive for most classes. However, it is also evident that the true positives of class B (Figure 12a) and class A (Figure 13a) are not improved.

**Figure 14.**
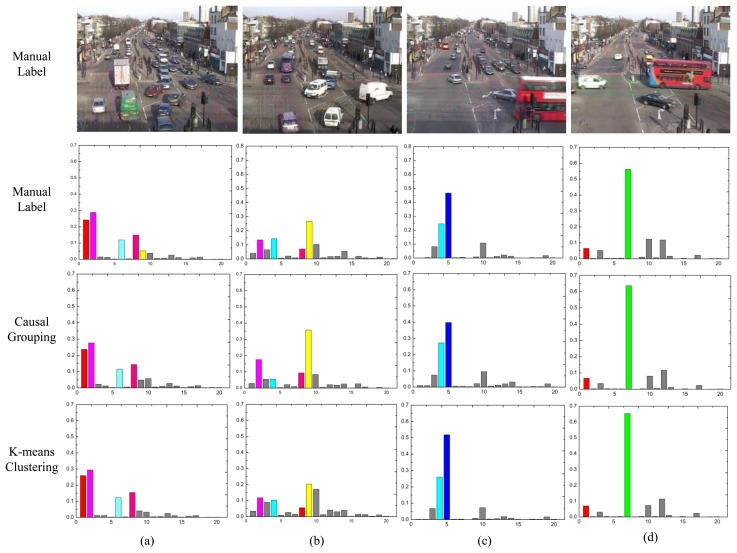
The topic distributions for the intersection dataset. The short video clips are grouped into four clusters. Different colors indicate the different topics respectively.

**Figure 15.**
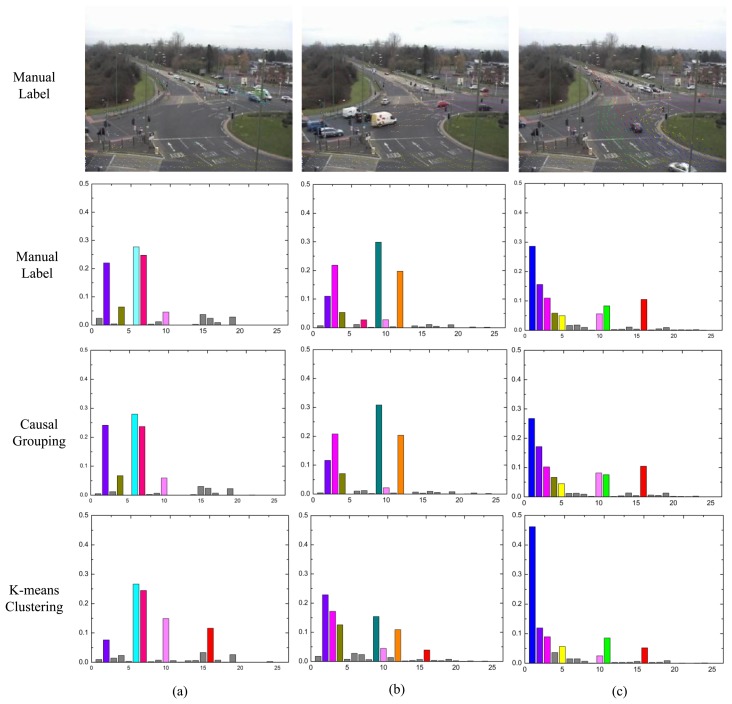
The topic distributions for the roundabout dataset. The short video clips are grouped into three clusters. Different colors indicate the different topics respectively.

To provide further insights on the performance difference, the distribution over the atomic topics for each class are analyzed. For the manual label and causal grouping, the average topic mixture of each class is computed (without sorting). However, for the K-means method, the clustering centers are chosen by itself. As shown in the first rows of Figures 14 and 15, the dominant topics for each class are illustrated by the most likely visual words. In the other three rows, the topic mixture proportions corresponding to each class are represented by bars. The x-axis is the index of atomic activities. The y-axis is the mixture over atomic activities. Different colors indicate the different topics respectively.

In Figure 14, (a) explains the traffic moving in a vertical direction; (b) represents turning traffic with various vertical traffic; (c) and (d) represent the rightward and leftward traffic flows, respectively. Clearly, topic 4 (topic 6 in Section 4.1) is shared among class B and C, but class B is only represented by topic 9 (topic 8 in Section 4.1); thus, it is easily misclassified as class C (see Figure 12a).

In Figure 15, (a) explains the leftward traffic flows; (d) represents the rightward traffic flows; (c) represents the vertical with right turning traffic flow. As the shown by the first row in Figure 15, topics 2, 4 and 10 (topics 2, 5 and 11 in Section 4.1) are independent of the other topics; they are always present. Furthermore, topic 3 (also topic 3 in Section 4.1) is shared by both class B and class C, but in Figure 10 class B does not include this topic. This trend results in a few real class B clips being misclassified as class C (see Figure 13a). Overall, the topic distribution for each class of the causal grouping method is more consistent with the manual label results, especially for class C.

## 5. Conclusions and Discussion

Scene analysis is a challenging problem in crowded outdoor environments, especially in situations where multiple activities are occurring simultaneously. In this paper, we present a novel framework to understand the complex scenes by exploring activity interactions and their temporal dependencies. First, a statistical denoising method is proposed to select useful dynamic regions in the scene for further analysis, and a codebook is generated using adaptive quantization. Next, we proposed an approach to interpret the atomic activities explored by the HDP model as multivariate point process. By performing the non-parameter Granger causal analysis on pairs of atomic activities, we can identify patterns of activity interactions and temporal rules. Additionally, the result of the causal analysis is used as a feature for scene classification which achieves high quality performance compared with the K-means clustering.

In future studies, additional experiments on different datasets will be conducted to evaluate the generalization of the proposed approach. Furthermore, the sensitivity to parameter settings is also a question that will be considered and investigated. Moreover, during the causality analysis, in addition to causal scores, the causal period should be considered to explore more exact topic interactions. We will study more precise causal grouping algorithms and better usage of the causal analysis results.

## Figures and Tables

**Table 1.** Causal matrix for the intersection dataset.

**Topic**	**1**	**2**	**3**	**4**	**5**	**6**	**7**	**8**
**1**	0.00	***0.93***	0.39	***0.80***	0.43	0.55	***0.62***	***0.63***
**2**	0.33	0.00	0.42	0.53	0.33	0.36	0.32	0.44
**3**	***0.61***	0.54	0.00	0.41	0.58	0.24	***0.68***	0.45
**4**	0.50	0.48	***1.87***	0.00	0.56	***1.04***	0.59	0.43
**5**	0.26	0.42	0.46	0.53	0.00	0.35	0.38	0.40
**6**	0.57	0.56	***1.33***	***0.72***	0.53	0.00	***0.63***	0.43
**7**	***0.63***	***1.64***	0.43	***0.71***	***0.60***	0.47	0.00	***0.65***
**8**	0.43	0.43	0.46	***0.67***	0.37	0.44	0.40	0.00

**Table 2.** Causal matrix for the roundabout dataset.

**Topic**	**1**	**2**	**3**	**4**	**5**	**6**	**7**	**8**	**9**	**10**	**11**	**12**
**1**	0.00	0.42	***0.74***	0.50	0.52	0.55	***0.79***	0.46	***2.20***	0.54	0.45	***1.17***
**2**	0.43	0.00	0.44	0.48	0.29	0.45	0.42	0.51	0.41	0.42	0.34	0.41
**3**	***0.74***	0.60	0.00	0.50	0.52	0.49	0.59	0.56	0.54	0.53	0.39	***0.71***
**4**	***0.84***	0.58	***2.21***	0.00	0.50	***4.42***	0.59	0.69	0.54	0.69	0.37	***0.83***
**5**	0.32	0.51	0.37	0.38	0.00	0.35	0.34	0.44	0.35	0.40	0.28	0.40
**6**	***0.87***	0.62	***2.60***	0.54	0.52	0.00	0.68	***0.76***	0.62	0.68	0.33	***0.99***
**7**	0.26	0.37	0.35	0.34	0.45	0.30	0.00	0.35	***0.90***	0.33	0.41	0.28
**8**	0.65	0.66	0.67	***0.72***	0.53	0.69	0.61	0.00	0.52	***1.75***	0.35	0.63
**9**	0.24	0.34	0.38	0.41	0.42	0.50	0.32	0.35	0.00	0.37	0.30	0.38
**10**	0.52	0.55	0.69	***0.72***	0.52	***0.75***	0.53	0.34	0.49	0.00	0.28	0.53
**11**	0.27	0.24	0.32	0.30	0.34	0.28	0.30	0.50	0.30	0.37	0.00	0.29
**12**	0.39	0.50	***1.23***	0.61	0.44	0.54	0.46	0.53	0.55	0.47	0.31	0.00

**Table 3.** Causal matrix for the subway dataset.

**Topic**	**1**	**2**	**3**	**4**	**5**	**6**	**7**	**8**	**9**	**10**	**11**
**1**	0.00	***1.47***	0.33	0.31	0.28	0.20	0.29	0.32	0.25	0.32	***0.89***
**2**	0.33	0.00	0.36	0.31	0.28	0.27	0.31	0.27	0.24	0.32	***1.45***
**3**	0.28	0.30	0.00	0.29	0.26	0.23	0.19	0.31	0.26	0.24	0.26
**4**	0.39	0.47	0.34	0.00	0.28	0.30	0.30	0.40	***0.55***	0.31	0.41
**5**	0.34	***0.61***	0.31	0.34	0.00	0.35	0.30	0.32	0.35	0.39	***0.52***
**6**	***0.81***	***1.02***	0.27	0.30	0.28	0.00	0.30	0.31	***0.60***	0.23	***0.86***
**7**	0.32	0.28	***0.69***	0.36	0.28	0.25	0.00	***0.79***	0.29	0.30	0.24
**8**	0.32	0.35	***0.66***	0.33	0.41	0.29	0.27	0.00	0.34	0.22	0.34
**9**	0.38	***0.75***	0.32	0.26	0.30	0.31	0.33	0.31	0.00	0.30	***0.73***
**10**	0.31	0.38	0.27	0.32	0.24	0.27	***0.59***	***0.53***	0.36	0.00	0.43
**11**	0.34	0.43	0.30	0.34	0.29	0.28	0.23	0.35	0.30	0.29	0.00

**Table 4.** The classification accuracy for the different quantization resolution.

**Quantization Resolution**	4*4	8*8	16*16	32*32	64*64	**Adaptive**
**Codebook size**	25,920	6480	1656	432	40	684
**Overall accuracy**	83.94%	83.04%	79.77%	76.40%	72.00%	87.07%

**Table 5.** The classification accuracy for the intersection dataset.

	**K-Means Clustering**	**Causal Grouping**
A	91.17%	98.59%
B	95.73%	64.02%
C	88.58%	96.30%
D	84.58%	87.75%
Overall	89.91%	91.16%

**Table 6.** The classification accuracy for the roundabout dataset.

	**K-Means Clustering**	**Causal Grouping**
A	96.49%	87.72%
B	80.00%	89.23%
C	52.85%	90.24%
Overall	70.20%	89.39%
